# Arbuscular mycorrhizal fungi alter the food utilization, growth, development and reproduction of armyworm (Mythimna separata) fed on *Bacillus thuringiensis* maize

**DOI:** 10.7717/peerj.7679

**Published:** 2019-09-12

**Authors:** Long Wang, Sabin Saurav Pokharel, Fajun Chen

**Affiliations:** Department of Entomology, Nanjing Agricultural University, Nanjing, China

**Keywords:** Arbuscular mycorrhizal fungi, Transgenic *Bt* maize, *Mythimna separata*, Food utilization, Growth & development and reproduction

## Abstract

**Background:**

The cultivation of *Bt* maize (maize genetically modified with *Bacillus thuringiensis*) continues to expand globally. Arbuscular mycorrhizal fungi (AMF), an important kind of microorganism closely related to soil fertility and plant nutrition, may influence the ecological risk of target lepidopteran pests in *Bt* crops.

**Methods:**

In this study, transgenic *Bt* maize (Line IE09S034 with *Cry1Ie* vs. its parental line of non-*Bt* maize cv. Xianyu335) was inoculated with a species of AMF, *Glomus caledonium* (GC). Its effects on the food utilization, reproduction and development of armyworm, *Mythimna separata*, were studied in a potted experiment from 2017 to 2018.

**Results:**

GC inoculation increased the AMF colonization of both modified and non-modified maize, and also increased the grain weight per plant and 1,000-grain weight of modified and non-modified maize. However, the cultivation of *Bt* maize did not significantly affect the AMF colonization. The feeding of *M. separata* with *Bt* maize resulted in a notable decrease in RCR (relative consumption rate), RGR (relative growth rate), AD (approximate digestibility), ECD (efficiency of conversion of digested food) and ECI (efficiency of conversion of ingested food) parameters in comparison to those observed in larvae fed with non-*Bt* maize in 2017 and 2018, regardless of GC inoculation. Furthermore, remarkable prolongation of larval life span and decreases in the rate of pupation, weight of pupa, rate of eclosion, fecundity and adult longevity of *M. separata* were observed in the *Bt* treatment regardless of GC inoculation during the two-year experiment. Also, when *M. separata* was fed with *Bt* maize, a significant prolongation of larval life and significant decreases in the pupal weight, fecundity and adult longevity of *M. separata* were observed when inoculated with GC. However, it was just the opposite for larvae fed with non-*Bt* maize that was inoculated with GC. The increased percentage of larval life-span, the decreased percentages of the food utilization, and the other indexes of reproduction, growth, and development of *M. separata* fed on *Bt* maize relative to non-*Bt* maize were all visibly lower when under GC inoculation in contrast to the CK.

**Discussion:**

It is presumed that *Bt* maize has a marked adverse impact on *M. separata* development, reproduction and feeding, especially when in combination with the GC inoculation. Additionally, GC inoculation favors the effectiveness of *Bt* maize against *M. separata* larvae by reducing their food utilization ability, which negatively affects the development and reproduction of the armyworm. Thus, *Bt* maize inoculated with AMF (here, GC) can reduce the severe threats arising of armyworms, and hence the AMF inoculation may play an important ecological functions in the field of *Bt* maize ecosystem, with potentially high control efficiency for the target lepidopteran pests.

## Introduction

The global cultivation of *Bt* maize (maize genetically modified with *Bacillus thuringiensis*) continues to expand globally (James, 2012). Transgenic *Bt* maize is one of the most produced genetically modified (GM) crops, and has been genetically engineered to express at least one insecticidal toxins derived from the soil bacterium *B. thuringiensis* (Alyokhin, 2011; Huang et al., 2014). These Cry (crystalline proteins) toxins from *B. thuringiensis* that affect a number of insect groups. At least 60 Cry proteins have been identified (Icoz & Stotzky, 2008; Sanchis, 2011). These insecticidal toxins (i.e., *Bt* toxins) help mitigate the damage done by some target lepidopteran insects (Walters et al., 2010; Pardo-López, Soberón & Bravo, 2013; Huang et al., 2014), such as *Ostrinia nubilalis*, *Ostrinia furnacalis* and *Mythimna separata* (Sanahuja et al., 2011; Guo et al., 2016; Jia et al., 2016).

Arbuscular mycorrhizal fungi (AMF) form symbiotic relationships with plant roots for the purpose of improving the uptake of water and nutrients, accelerating plant growth, and helping to build soil structure and function (Smith & Read, 2008). Correspondingly, AMFs require an adequate plant host. The fungi get carbon from plants, and in return, fungi give nitrogen, phosphorus and other nutrients, and can improve soil stability and resistance to disease (Singh, Singh & Tripathi, 2012; Steinkellner et al., 2012). Plants associated with AMF may alter their interactions with insects, pollinators or herbivores, and this will affect the plant health (Vannette & Rasmann, 2012; Koricheva & Jones, 2009; Wolfe, Husband & Klironomos, 2005). AMF colonization often affects insect herbivores (Koricheva & Jones, 2009), AMF influences defense chemicals, nutrient contents, and plant biomass (Bennett, Bever & Bowers, 2009). For instance, the interaction with mycorrhizal fungi may give the plant resources against hervibores, but it may instead make the plant a better food source (Vannette & Hunter, 2011).

Food nutrition is an important indicator of insect selection behavior and food competition behavior. The choice of insects for different foods is related to the efficiency of insects utilization of food. Different foods directly affect the growth and development of insects and the efficiency of food utilization (Schmidt et al., 2012). Hence, Waldbauer (1968) suggested using RCR (relative consumption rate), RGR (relative growth rate), AD (approximate digestibility), ECD (efficiency of conversion of digested food) and ECI (efficiency of conversion of ingested food) as nutritional indicators to measure the efficiency of food digestion. Moreover, previous research has shown nitrogen is the most active nutrient element in the crop growth process and is the main constituent of *Bt* protein. Plant nitrogen uptake and nitrogen metabolism levels can change the carbon-nitrogen ratio in plant tissues and can also affect the production of *Bt* toxins (Jiang et al., 2013; Gao et al., 2009). The presence of *Bt* toxin also affects insect’s feeding efficiency, growth and reproduction (Li, Parajulee & Chen, 2018). AMF can enhance plant absorption and utilization of soil nutrients (mainly N and P). Thus, the effects of AMF on *Bt* maize and target lepidopteran pests has naturally become an interesting and significant research priority. There have been some studies that focused on the influence of *Bt* on AMF colonization, the AMF community diversity, and soil ecology (Zeng et al., 2014; Cheeke, Cruzan & Rosenstiel, 2013). However, the effects of AMF on the resistance of *Bt* crops against target lepidopteran pests has not been explored in the previous reports. In this work, we studied the indirect effects of AMF on the food utilization, growth, and development of armyworm *M. separata* feeding on transgenic *Bt* maize, and the direct influence of AMF on the yields of *Bt* and non-*Bt* maize. We expect that this work will help reduce the risk of *Bt* crops resistance and ultimately provide for the sustainable and ecological usage of *Bt* crops.

## Materials and Methods

### Plant materials and AMF inoculation

A two-year study (2017–2018) was conducted in Ningjin County, Shandong Province of China (37°38′30.7″N, 116°51′11.0″E). The Institute of Crop Sciences, Chinese Academy of Agricultural Science provided us with the transgenic *Bt* maize cultivar (Line IE09S034 with *Cry1Ie*, Bt) and its non-*Bt* parental line (cv. Xianyu 335, Xy). *Glomus caledonium* (strain number 90036, referred to as GC) was provided by the State Key Laboratory of Soil & Sustainable Agriculture, Institute of Soil Science, Chinese Academy of Sciences. The inoculum consisted of spores, mycelium, maize root fragments, and soil. Both genetically modified and non-modified maize were put into plastic buckets (45 cm height, 30 cm diameter) with 20 kg of soil sterilized in an autoclave, 300 g GC inoculum (GC inoculation treatment, ab. GC) and 300 g sterilized strains (control group, ab. CK) were evenly spread four cm below the maize seeds on June 10 in each sampling year. The whole experiment involved four treatments: two maize cultivars (Bt and Xy), and two AMF inoculations (GC and CK). Each bucket served as one replication and replicated 15 times for each treatment. So, there were 15 buckets for each maize cultivar × AMF inoculation treatment, and a total of 60 buckets in this study. In each bucket, three maize seeds were put at a depth of two cm. During the whole experimental period, no pesticides were applied and the manual weeding was performed to keep the maize buckets free from incidence of weeds.

### AMF colonization

AMF colonization was determined on July 3 (seeding stage), August 25 (heading stage) and September 23 (harvest stage) in two sampling years. This was determined by the method of trypan blue staining and grid counting (Phillips & Hayman, 1970). The fresh plant roots were washed with distilled water and then blotted dry with absorbent paper. One hundred one cm roots were randomly cut and placed in a 10% KOH solution at 30 °C for 30 min, and then the KOH was discarded and rinsed with distilled water. After acidification in 2% HCl for 60 min, the HCl was discarded, rinsed with distilled water and stained in 5% trypan blue dye solution (w/v, lactic acid: glycerol: water = 1:1:1). Then the dye solution was discarded, and the roots were rinsed with distilled water and transferred to a square with a grid at the bottom. We observed the number of infected and uninfected root segments under the microscope. Colonization (%) = number of infected root segments/total root segments (McGonigle et al., 1990).

### Insect rearing

The colony of armyworm *M. separata* was originated from a population collected in maize fields in Kangbao County, Hebei province of China (41.87°N, 114.6°E) in the summer of 2014. They were reared on the artificial diet (Bi, 1981) for more than 15 generations in climate-controlled growth chambers (GDN-400D-4; Ningbo Southeast Instrument Co., Ltd., Ningbo, China) at 26  ± 1 °C, 65  ± 5% RH, and 14: 10 h L/D photoperiod. The same rearing breeding conditions were maintained kept for the subsequent experiments. The newly-hatched first instar larvae were randomly selected from the above colony of *M. separata* and fed on the same artificial diet until the second instar larval stage, and then the third instar *M. separata* larvae were individually fed on excised leaves of the sampled maize plants. A certain amount of experimental maize leaves were randomly chosen from 10 buckets of each maize cultivar × AMF inoculation treatment beginning August 22 (heading stage) for the feeding trials conducted in plastic dish (six cm in diameter and 1.6 cm in height) and replace fresh maize leaves every 24 h until *M. separata* pupation in 2017 and 2018. Each maize cultivar × AMF inoculation treatment consisted of five replicates (30 larvae for one replicates).

### Food utilization of *M. separata* larvae

The initial weights of the tested third instar larvae of *M. separata* were individually determined with an electronic balance (AL104; METTLER-TOLEDO, Greifensee). The weights of the total feces from the third instar until pupation (sixth instar), pupal weight, and the residual leaves were also carefully measured. At the same time, the moisture content of the third instar larvae, the sixth instar larvae and maize leaves replaced each time were determined to calculate the dry weight of the tested larvae and the maize leaves during the experiment. Several food utilization indexes of *M. separata* larvae fed on the excised leaves of *Bt* and non-*Bt* maize inoculated with AMF, *G. caledonium* and without *G. caledonium*, were determined. The indexes included RCR (relative consumption rate), RGR (relative growth rate), AD (approximate digestibility), ECD (efficiency of conversion of digested food) and ECI (efficiency of conversion of ingested food) (Li, Parajulee & Chen, 2018). The indexes calculations were done with formulas adapted from Chen et al. (2005):

\begin{eqnarray*}& & \mathrm{RCR}=I/(B\ast T); \mathrm{RGR}=G/(B\ast T); \mathrm{AD}  \left( \text{%} \right) =(I-F)/I\ast 100\text{%}; \end{eqnarray*}

\begin{eqnarray*}& & \mathrm{ECD}  \left( \text{%} \right) =G/(I-F)\ast 100\text{%}; \mathrm{ECI}  \left( \text{%} \right) =G/I\ast 100\text{%}. \end{eqnarray*}

Where *I* is the feeding amount (the dry weight of maize leaves before feeding minus the dry weight of maize leaves before feeding after feeding); *B* is the average larval weight during the experiment (the average larval dry weight before feeding and after feeding); *T* is experiment time (d); *G* is the added larval weight (the larval dry weight after feeding minus the larval dry weight before feeding); *F* is the dry weight of total feces.

### Growth & development and reproduction of *M. separata*

Larval growth and development were evaluated from the third instar to pupation by observing each petri dish every 8 h and recording the timing of larval ecdysis, pupation, and emergence of *M. separata* moths that fed on the excised leaves of *Bt* and non-*Bt* maize inoculated with *G. caledonium* and without *G. caledonium*. After the eclosion, novel moths were paired by maintaining the female: male ratio of 1: 1 in a metal screen cage and were fed with a 10% honey cotton ball, covered with cotton net yarn and butter paper for oviposition which were replaced every day. Survivorship and oviposition were recorded on a daily basis until death.

### Yield of *Bt* and non-*Bt* maize

On September 25, 2017 and 2018, eight maize plants were randomly taken from five pots of each maize cultivar × AMF inoculation treatment at the harvest stage to measure the grain weight per plant (g) and 1,000-grain weight (g) with an electronic balance (AL104; METTLER-TOLEDO, Greifensee, Switzerland), in order to ascertain the effects of AMF inoculation on the yield of *Bt* and non-*Bt* maize inoculated with and without GC.

### Data analysis

All experimental data were analysed with the software IBM-SPSSv.20.0 (IBM, Armonk, NY, USA). Three-way repeated-measures ANOVA was used to study the impacts of treatment (*Bt* maize vs. non-*Bt* maize), AMF inoculation (GC vs. CK), sampling years (2017 vs. 2018), and their bi- and tri-interaction on the AMF colonization. Moreover, three-way ANOVA was used to analyze the effects of treatment (*Bt* maize vs. non-*Bt* maize), AMF inoculation (GC vs. CK), sampling years (2017 vs. 2018), and their bi- and tri-interactions on the measured indexes of growth, development, reproduction and food utilization of *M. separata*, and the yield of *Bt* and non-*Bt* maize inoculated with and without GC in 2017 and 2018. Finally, the means were separated by using the Turkey test to examine significant difference between/among treatments at *P* < 0.05.

## Results

### AMF colonization of *Bt* and non-*Bt* maize inoculated with and without *G. caledonium*

**Figure 1:**
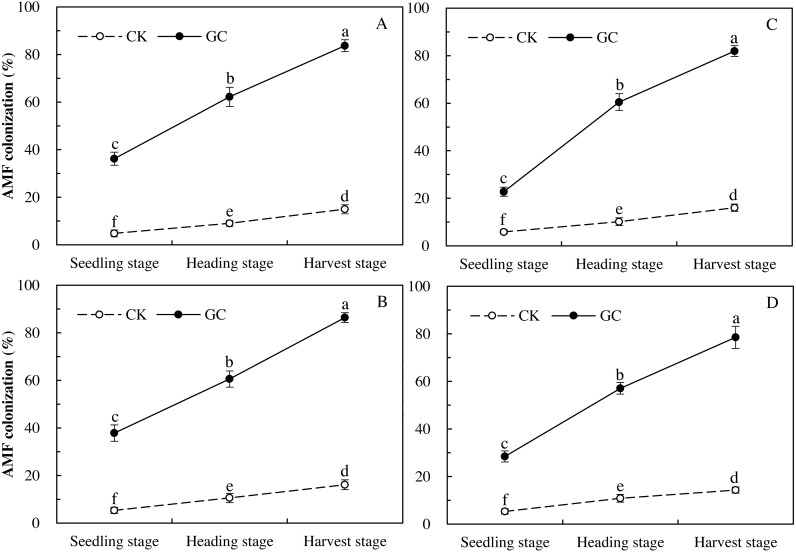
AMF colonization dynamics of transgenic *Bt* maize (A and C) and its parental line of non-*Bt* maize (B and D) inoculated with and without *G. caledonium* in 2017 (A and B) and 2018 (C and D). GC represents *G. caledonium* inoculation treatment; CK represents control group. Different lowercase letters indicate significant differences between treatments of GC inoculation and the control group with maize growth stages as repeated measures in the same sampling year by the Tukey test at *P* < 0.05.

Colonization represents the infestation status of inoculated AMF, proving whether the access of AMF in maize is effective. Three-way repeated-measures ANOVAs indicated that GC inoculation (*P* < 0.001) and sampling year (*P* < 0.001) both significantly affected the AMF colonization, and there were significant interactions between GC inoculation with sampling year (*P* < 0.001), and between transgenic treatment with sampling year (*P* = 0.013 < 0.05; Table 1). Compared with the non-GC inoculation, the GC inoculation significantly enhanced the AMF colonization of *Bt* and non-*Bt* maize in 2017 and 2018 respectively, with significant increases for the *Bt* maize during the seedling (2017: +653.9%; 2018: +284.1%), heading (2017: +589.6%; 2018: +491.0%) and harvest (2017: +457.6%; 2018: +409.6%) stages in 2017 and 2018, and for the non-*Bt* maize during the seedling (2017: +613.6%; 2018: +432.2%), heading (2017: +472.8%; 2018: +425.7%) and harvest (2017: +437.1%; 2018: +448.7%) stages in 2017 and 2018 (*P* < 0.05; Fig. 1).

**Table 1:** Three-way repeated-measures ANOVAs on the AMF colonization and three-way ANOVAs on the yields of *Bt* and non-*Bt* maize inoculated with and without *G. caledonium* in 2017 and 2018 (*F*/*P* values).

Factors	Colonization (%)	Grain weight per plant (dry; g)	1,000-grain weight (dry; g)
Y^a^	70.10/<0.001^***^	90.57/<0.001^***^	0.05/0.83
Cv.^b^	0.49/0.49	0.23/0.64	5.87/0.028^*^
AMF^c^	6673.63/<0.001^***^	144.17/<0.001^***^	92.54/<0.001^***^
Y × Cv.	7.80/0.013^*^	4.96/0.041^*^	17.96/0.001^**^
Y × AMF	48.56/<0.001^***^	8.11/0.012^*^	0.12/0.73
Cv. × AMF	0.04/0.85	0.01/0.92	0.14/0.71
Y × Cv. × AMF	0.02/0.91	2.10/0.17	0.30/0.59

**Notes.**

* *P* < 0.05.

** *P* < 0.01.

*** *P* < 0.001.

a Year (2017 vs. 2018).

b Transgenic treatment (*Bt* maize vs. non-*Bt* maize).

c AMF inoculation (GC *vs.* CK).

### Food utilization of *M. Separata* larvae fed on *Bt* and non-*Bt* maize inoculated with and without *G. caledonium*

Food utilization indexes can reflect the preference and adaptability of insects to food materials to a certain extent. Transgenic treatment significantly affected all the measured indexes of feeding of *M. separata* larvae (*P* < 0.001), AMF inoculation (*P* < 0.05) and the interactions between transgenic treatment and AMF inoculation (*P* < 0.001) had important effects on the RGR, RCR, ECI and AD of *M. separata* larvae, and there were significant differences in the RGR, ECD, ECI and AD of *M. separata* larvae between the two sampling years (*P* < 0.01; Table 2). Moreover, there were significant interactions between transgenic treatment and sampling year on the RGR, RCR and AD of *M. separata* larvae (*P* < 0.05; Table 2). Furthermore, there were significant interactions between AMF inoculation and sampling year, and among transgenic treatment, AMF inoculation and sampling year on the RGR of *M. separata* larvae fed on the detached leaves of *Bt* maize and its parental line of non-*Bt* maize during the heading stage in 2017 and 2018.

**Figure 2:**
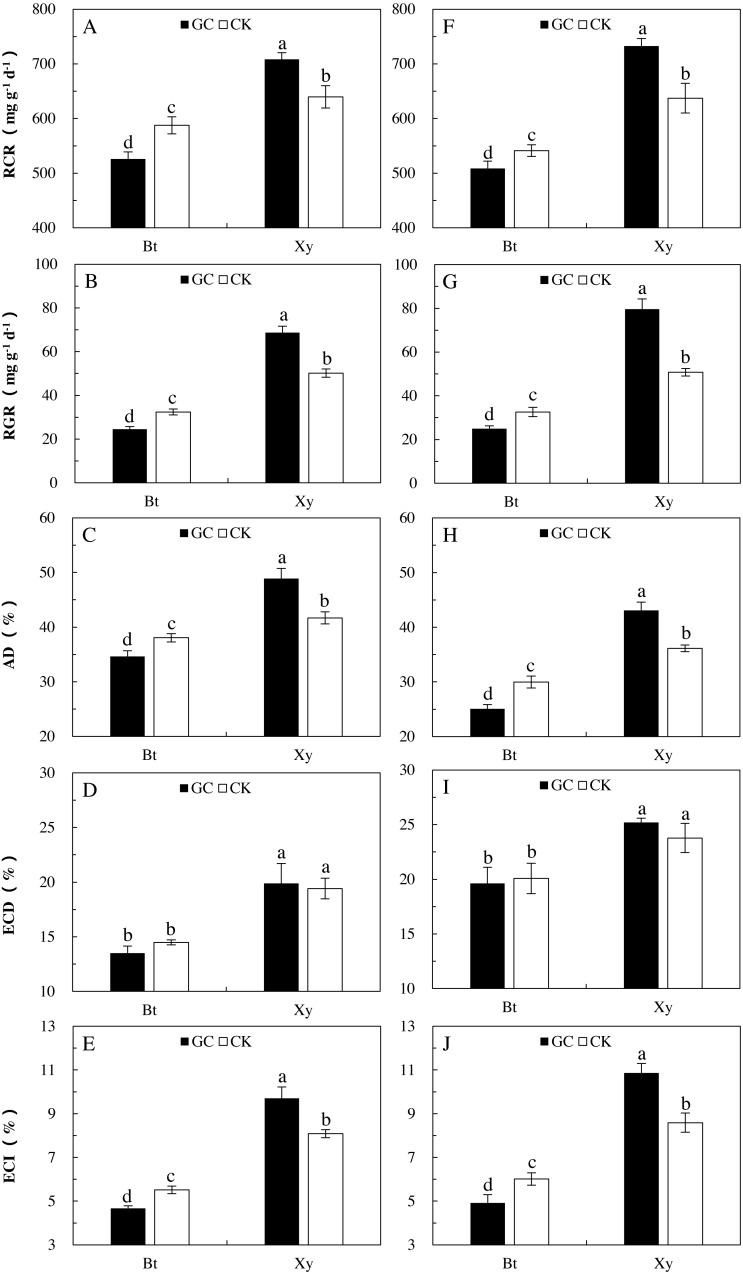
Food utilization of *M. separata.* from the third to the sixth instar larvae fed on *Bt* maize (Bt) and non-*Bt* maize (Xy) inoculated with and without *G. caledonium* in 2017 (A–E) and 2018 (F–J). GC represents *G. caledonium* inoculation treatment; CK represents control group. A and F, RCR (relative consumption rate); B and G, RGR (relative growth rate); C and H, AD (approximate digestibility); D and I, ECD (efficiency of conversion of digested food); E and J, ECI (efficiency of conversion of ingested food). Different lowercase letters indicate significant differences between treatments in same sampling year by the Turkey test at *P* < 0.05.

When considering the case of *M. separata* larvae fed on *Bt* maize and non-*Bt* maize inoculated with GC in comparison with the CK in both sampling years, differing trends in the food utilization indexes were observed (Fig. 2). In relation to the CK (i.e., non-GC inoculation), GC inoculation significantly reduced the RGR (−24.2% and −23.3%), RCR (−10.5% and −6.1%), ECI (−15.3% and −18.2%) and AD (−9.0% and −16.4%) of *M. separata* larvae fed on the detached leaves of *Bt* maize during the heading stage in 2017 and 2018 (*P* < 0.05; Fig. 2). However, GC inoculation significantly enhanced the RGR (+36.9% and +56.7%), RCR (+10.8% and +15.0%), ECI (+19.9% and +26.4%) and AD (+17.2% and +19.3%) of *M. separata* larvae fed on the detached leaves of non-*Bt* maize during the heading stage in 2017 and 2018 (*P* < 0.05; Fig. 2).

Moreover, significant decreases in the RGR (2017: −64.2% and −35.4%; 2018: −68.6% and −35.9%), RCR (2017: −25.7% and −8.1%; 2018: −30.6% and −15.1%), ECD (2017: −32.2% and −25.4%; 2018: −22.1% and −15.5%), ECI (2017: −51.9% and −31.8%; 2018: −54.7% and −30.0%), and AD (2017: −29.2% and −8.7%; 2018: −41.8% and −17.1%) were found when *M. separata* larvae fed on the detached leaves of *Bt* maize in contrast to non-*Bt* maize as inoculated with and without GC in 2017 and 2018 (*P* < 0.05; Fig. 2). Additionally, the decreased percentages in the RGR, RCR, ECD, ECI and AD of *M. separata* larvae fed on the detached leaves of *Bt* maize were all obviously higher under GC inoculation in contrast to CK when compared with non-*Bt* maize.

**Table 2:** Three-way ANOVAs on the food utilization of *M. separata* from the 3rd to the 6th instar larvae fed on *Bt* and non-*Bt* maize inoculated with and without *G. caledonium* in 2017 and 2018 (*F/P* values).

Factors	RGR (mg g^−1^d^−1^)	RCR (mg g^−1^d^−1^)	ECD (%)	ECI (%)	AD (%)
Y^a^	9.49/0.007^**^	2.44/0.14	128.14/<0.001^***^	18.04/0.002^**^	222.90/<0.001^***^
Cv.^b^	1176.29/<0.001^***^	418.12/<0.001^***^	990.94/<0.001^***^	804.52/<0.001^***^	485.47/<0.001^***^
AMF^c^	65.72/<0.001^***^	6.60/0.021^*^	0.06/0.82	11.67/0.004^**^	9.20/0.008^**^
Y × Cv.	7.83/0.013^*^	10.06/0.006^**^	1.20/0.29	2.61/0.13	11.01/0.004^**^
Y × AMF	7.19/0.016^*^	4.16/0.058	0.61/0.45	0.51/0.49	0.80/0.39
Cv. × AMF	303.71/<0.001^***^	91.07/<0.001^***^	3.13/0.096	104.96/<0.001^***^	138.78/<0.001^***^
Y × Cv. × AMF	2.58/0.021^*^	0.01/0.94	0.05/0.83	2.54/0.13	0.44/0.52

**Notes.**

* *P* < 0.05.

** *P* < 0.01.

*** *P* < 0.001.

a Year (2017 vs. 2018).

b Transgenic treatment (*Bt* maize vs. non-*Bt* maize).

c AMF inoculation (GC *vs.* CK).

### Reproduction, growth and development of *M. Separata* fed on *Bt* or non-*Bt* maize inoculated with and without *G. caledonium*

The effects of a *Bt* and non-*Bt* maize diet on the growth, development and reproduction of *M. Separata* demonstrate the indirect effects of AMF on the suitability of *M. Separata* through *Bt* and non-*Bt* maize. Transgenic treatment (*P* < 0.05) and AMF inoculation (*P* < 0.05) significantly affected all the calculated indexes of *M. separata* in two sampling years, and there were significant difference in pupal weight (*P* < 0.001) of *M. separata* between two sampling years (Table 3). Moreover, there were significant interactions between transgenic treatment and sampling year on larval life-span (*P* = 0.008 < 0.01), between AMF inoculation and sampling year on larval life-span (*P* = 0.011 < 0.05), adult longevity (*P* = 0.044 < 0.05) and fecundity (*P* = 0.004 < 0.01), between transgenic treatment and AMF inoculation on all the measured indexes except larval life-span (*P* < 0.01), and among transgenic treatment, AMF inoculation and sampling year on larval life-span (*P* = 0.013 < 0.05) and fecundity (*P* = 0.009 < 0.01) for *M. separata* fed on the detached leaves of *Bt* maize and non-*Bt* maize inoculated with and without GC during the heading stage in 2017 and 2018 (Table 3).

**Figure 3:**
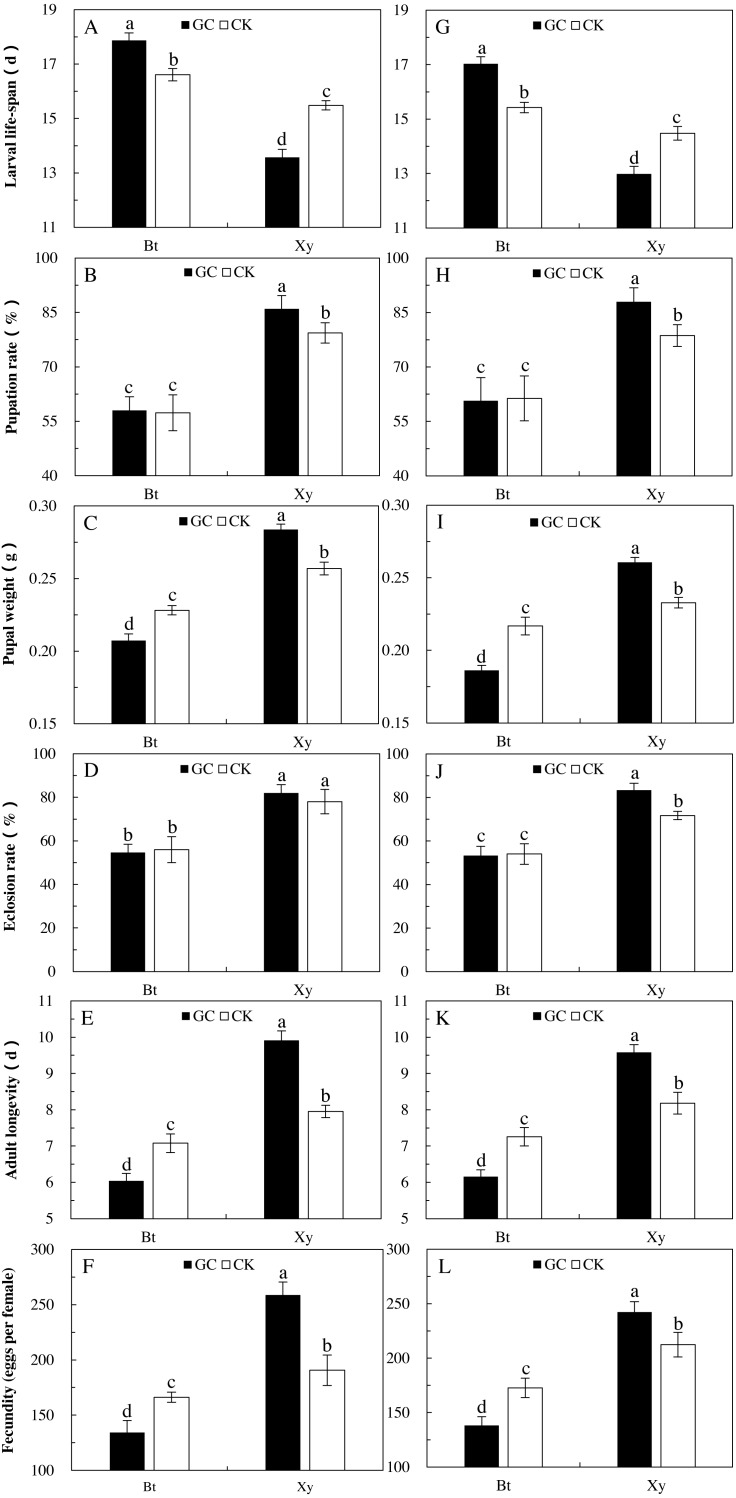
Growth, development and reproduction of *M. separata* fed on *Bt* maize (Bt) and non-*Bt* maize (Xy) inoculated with and without *G. caledonium* in 2017 (A–F) and 2018 (G–L). GC represents *G. caledonium* inoculation treatment; CK represents control group. A and G, Larval life-span; B and H, Pupation rate; C and I, Pupal weight; D and J, Eclosion rate; E and K, Adult longevity; F and L, Fecundity. Different lowercase letters indicate significant differences between treatments in same sampling year by the Tukey test at *P* < 0.05.

Opposite trends were also seen in the calculated indexes for reproduction, growth, and development of larvae fed on the detached leaves of *Bt* and non-*Bt* maize inoculated with GC in contrast to the CK in both sampling years (Fig. 3). In comparison with the CK, GC inoculation significantly extended the larval life cycle (+7.6% and +10.4%) and shortened the adult longevity (−14.7% and −15.2%), and decreased the pupal weight (−9.1% and −14.1%) and fecundity (−19.2% and −19.9%) of larvae fed on the detached leaves of *Bt* maize in 2017 and 2018 (*P* < 0.05; Fig. 3). At the same time, GC inoculation significantly shortened the larval life-span (−12.3% and −10.3%) and prolonged the adult longevity (+24.6% and +17.1%), and significantly increased the pupation rate (+8.4% and +11.9%), pupal weight (+10.5% and +11.9%) and fecundity (+35.7% and +14.1%) of larvae fed on the detached leaves of non-*Bt* maize in 2017 and 2018, and significantly increased the eclosion rate (+16.3%) of larvae fed on the detached leaves of non-*Bt* maize in 2018 (*P* < 0.05; Fig. 3).

**Table 3:** Three-way ANOVAs on the growth, development and reproduction of *M. separata* fed on *Bt* and non-*Bt* maize inoculated with and without *G. caledonium* in 2017 and 2018 (*F*/*P* values).

Factors	Larval life-span (day)	Pupation rate (%)	Pupal weight (g)	Eclosion rate (%)	Adult longevity (day)	Fecundity (eggs per female)
Y^a^	3.34/0.077	1.97/0.17	19.11/<0.001^***^	2.58/0.12	0.40/0.53	1.54/0.22
Cv.^b^	6.64/0.015^*^	275.83/0.008^**^	5.42/0.026^*^	320.94/<0.001^***^	934.67/<0.001^***^	516.08/<0.001^***^
AMF^c^	6.15/0.019^*^	7.87/0.008^**^	5.30/0.028^*^	6.73/0.014^*^	16.68/<0.001^***^	6.01/<0.020^*^
Y × Cv.	8.06/0.008^**^	0.87/0.36	1.52/0.23	0.14/0.71	1.80/0.19	0.14/0.71
Y × AMF	7.44/0.011^*^	0.06/0.82	0.51/0.48	2.58/0.12	4.39/0.044^*^	9.82/0.004^**^
Cv. × AMF	0.10/0.75	7.87/0.008^**^	27.38/<0.001^***^	11.13/<0.002^**^	341.13/<0.001^***^	162.70/<0.001^***^
Y × Cv. × AMF	6.91/0.013^*^	0.49/0.49	0.64/0.43	1.85/0.18	2.82/0.10	7.63/0.009^**^

**Notes.**

* *P* < 0.05.

** *P* < 0.01.

*** *P* < 0.001.

a Year (2017 vs. 2018).

b Transgenic treatment (*Bt* maize vs. non-*Bt* maize).

c AMF inoculation (GC *vs.* CK).

In comparison with the non-*Bt* maize, *Bt* maize significantly prolonged the larval life cycle (2017: +31.6% and +7.3%; 2018: +31.1% and +6.6%) and shortened the adult longevity (2017: −44.2% and −17.7%; 2018: −32.6% and −13.4%), and significantly decreased the pupation rate (2017: −32.6% and −27.7%; 2018: −31.1% and −22.0%), pupal weight (2017: −26.9% and −11.2%; 2018: −28.5% and −6.9%), eclosion rate (2017: −33.3% and −28.2%; 2018: −36.0% and −24.6%) and fecundity (2017: −48.1% and −12.8%; 2018: −43.0% and −18.7%) of larvae fed on the detached maize leaves inoculated with and without GC in 2017 and 2018 (*P* < 0.05; Fig. 3). Additionally, the larval life-span percentage increased, and percentages decreased in the pupation rate, pupal weight, eclosion rate, adult longevity and fecundity of larvae fed on the detached leaves of *Bt* maize were all obviously higher under GC inoculation in contrast to CK when compared with non-*Bt* maize.

### Yields of *Bt* and non-*Bt* maize inoculated with and without *G. caledonium*

The increase in yields is our ultimate goal; that is, the inoculation of AMF has a corresponding indirect effects on the *M. Separata* feeding on *Bt* and non-*Bt* maize, and in this section, we evaluated the change of the final economic maize output after inoculation. AMF inoculation and sampling year significantly affected the grain weight per plant (*P* < 0.001), and 1,000-grain weight were significantly affected by AMF inoculation and transgenic treatment (*P* < 0.05). There were significant interactions between sampling year and transgenic treatment on the grain weight per plant (*P* = 0.041 < 0.05) and 1,000-grain weight (*P* = 0.001 < 0.01), and between sampling year and AMF inoculation on the grain weight per plant (*P* = 0.012 < 0.05) of *Bt* and non-*Bt* maize inoculated with and without GC inoculation in 2017 and 2018 (Table 1).

**Figure 4:**
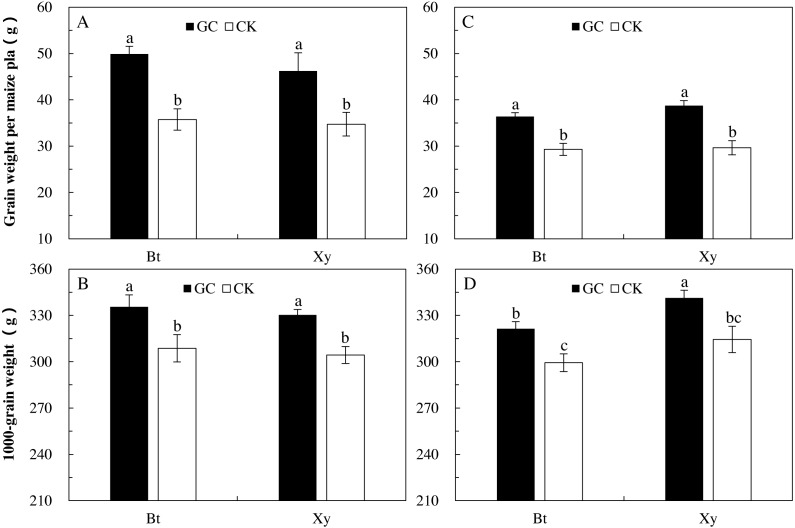
Grain weight per plant (A and C) and 1,000-grain weight (B and D) of *Bt* maize (Bt) and its parental line of non-*Bt* maize (Xy) inoculated with *G. caledonium* in 2017 (A and B) and 2018 (C and D). GC represents *G. caledonium* inoculation treatment; CK represents control group. Different lowercase letters indicate significant differences between treatments in same sampling year by the Tukey test at *P* < 0.05.

Compared with the CK, GC inoculation significantly increased the grain weight per plant (*Bt* maize: +39.6% and +24.1%; non-*Bt* maize: +33.1% and +30.6%) and 1,000-grain weight (*Bt* maize: +8.7% and +7.4%; non-*Bt* maize: +8.5% and +8.5%) of *Bt* and non-*Bt* maize in 2017 and 2018 (*P* < 0.05; Fig. 4).

## Discussion

AMF are a group of fungi belonging to phylum Glomeromycota that penetrates the cortex of the roots of vascular plants (Parniske, 2008; Smith & Read, 2008). On the whole, the colonization in 2018 was lower than 2017, this may be due to an increase of hot weather during the experiment in 2018 that unfavorable to colonization of AMF. However, despite the differences between the data from the two years, the impact trend of AMF inoculation was consistent. GC inoculation significantly enhanced AMF colonization of *Bt* and non-*Bt* maize from the seedling stage to the harvest stage in two sampling years, and this result demonstrated the effectiveness for the inoculation of AMF. No significant difference was found in 2017 or 2018 for the AMF colonization of both types of maize (*Bt* or non-*Bt*). So, it is presumed that the cultivation of *Bt* maize has no significant impact on the AMF colonization between *Bt* maize (Line IE09S034) expressing *Cry1Ie* protein and the near isogenic non-*Bt* variety (cv. Xianyu335). Although an important negative effect of *Bt* on the AMF community was found (Castaldini et al., 2005; Turrini et al., 2005), some researchers reached the conclusion that the cultivation of *Bt* crops had no significant impact on AMF colonization of roots between *Bt* maize (MEB307) expressing Cry1Ab protein and the near isogenic non-*Bt* variety (Monumental), and the arrangements of AMF in roots in non-*Bt* were almost identical to those in *Bt* cultivars of cotton (Cry1Ac and Cry2Ab) (Vaufleury et al., 2007; Knox et al., 2008).

Hodge, Helgason & Fitter (2010) reported that the AMF promoted the absorption and utilization of soil nutrients in maize plants, thus improving the nutrient levels of nitrogen, phosphorus and potassium of plant tissues and organs, and in turn, promoting the growth and development of maize plants. In our study, yield data also substantiated this viewpoint. The GC inoculation significantly increased the grain weight per plant and 1,000-grain weight regardless whether the maize was *Bt* or non-*Bt*, and this is exactly because of the promotion of plant nutrition by AMF. Meanwhile, there were no notable differences found in the grain weight per plant of *Bt* maize when compared with that of non-*Bt* maize, regardless of GC inoculation or non-GC inoculation. This result was also consistent with our results of colonization which showed that *Bt* treatment had no effect on AMF infection and that there was no difference in yields with its parental line of non-*Bt* maize.

Globally, transgenic *Bt* maize has been rapidly commercialized to control lepidopteran insects (for example: *Ostrinia nubilalis*, *Mythimna separata* and *Ostrinia nubilalis*) (James, 2012; ISAAA, 2017), but have been no reports that the defense responses of *M. separata* to transgenic *Bt* maize inoculated with AMF. Most studies have shown that Cry proteins have adverse effects on the life-table parameters of different herbivores (Lawo, Wäckers & Romeis, 2010; Smith & Fischer, 1983). Li, Parajulee & Chen (2018) reported that *Bt* maize significantly affected the food utilization, reproduction, growth & development of the armyworm, *M. separata*. The research of Prutz & Dettner (2005) showed that *Bt* maize decreased the rate of growth and increased the mortality of *Chilo partellus*. In this study, important reductions in the RCR, RGR, AD and ECI occurred when the larvae were fed on *Bt* maize inoculated with and without GC in 2017 and 2018. Moreover, *Bt* maize also markedly extended the larval life-span, shortened the adult longevity, and significantly decreased the pupation rate, pupal weight, eclosion rate and fecundity of larvae regardless of if they were inoculated with or without GC in 2017 and 2018. This can prove that *Bt* toxins protein does have a marked negative effects on the food utilization, reproduction, growth, and development of *M. separata.*

Opposite trends were found in the food utilization, reproduction, growth, and development of *M. separata* fed on *Bt* maize and non-*Bt* maize inoculated with and without GC. For the measured indexes of food utilization, GC inoculation significantly reduced the RGR, RCR, AD and ECI of larvae fed on *Bt* maize, while it was just the opposite was shown for those of larvae fed on non-*Bt* maize in 2017 and 2018. For the measured indexes of growth, development and reproduction, there were also opposite trends for larvae fed on *Bt* and non-*Bt* maize inoculated with and without GC. GC inoculation markedly extended the larval life-span, shortened the adult longevity, and significantly decreased the pupal weight and fecundity of fed on *Bt* maize in 2017 and 2018. Conversely, GC inoculation significantly shortened the larval life-span, prolonged the adult longevity, and significantly increased the pupation rate, pupal weight and fecundity of *M. separata* fed on non-*Bt* maize in 2017 and 2018, and significantly increased the eclosion rate of *M. separata* fed on non-*Bt* maize in 2018. This phenomenon can be explained by the fact that AMF inoculation promoted the absorption and utilization of soil nutrients in maize plants, thus improving the nutrient level (e.g., nitrogen, phosphorus and potassium) of plant leaves Hodge, Helgason & Fitter, 2010; Rodriguez & Sanders, 2015). Many studies have shown that the nitrogen metabolism and nitrogen level of transgenic *Bt* crops could affect the expression of *Bt* toxin protein (Wang et al., 2012; Liu et al., 2019). Stimulating in plant N uptake can increase biomass N relative to C and enhance the nitrogen metabolism enzyme (e.g., nitrate reductase, nitrite reductase, and so forth) activity, transgene expression and *Bt* toxin production of *Bt* crops (Stitt & Krapp, 1999; Gao et al., 2009; Jiang et al., 2017). In brief, AMF could enhance the maize nitrogen level which was important for *Bt* protein synthesis, therefore, the inoculation with AMF was beneficial to the production of *Bt* protein. For non-*Bt* maize, the leaf food source with high nutrition means the intake and utilization of high nutrient elements, which naturally has a more positive and beneficial effect on *M. separata*. For *Bt* maize, an increase in nutrient levels may also mean an increase in toxin protein expression, as higher toxins were bound to be more damaging to *M. separata*, which could account for the inverse trends in RGR, RCR, AD, and ECI fed on *Bt* maize and non-*Bt* maize inoculated with GC.

In addition, the increased percentage of the larval life-span, and decreased percentages of the indexes of food utilization, pupation rate, pupal weight, eclosion rate, adult longevity and fecundity of *M. separata* larvae fed on *Bt* maize when compared with non-*Bt* maize were obviously higher under the GC inoculation in contrast to CK. This is mainly due to the opposite impacts above-mentioned that AMF treatment appears on *M. separata* fed on *Bt* and non-*Bt* maize. AMF treatment is more beneficial for *M. separata* fed on non-*Bt* maize, but is more unfavorable for *M. separata* fed on *Bt* maize. This result indicates that *Bt* maize will have better control effects on *M. separata* when combined with GC inoculation.

## Conclusion

AMF can induce changes in plant morphology, physiology, biochemistry, and even gene expression, which in turn may change the food quality of herbivorous insects, thus affecting their feeding tendency, growth, reproduction and harmfulness (Jung et al., 2012). This research indicated that the inoculation of AMF using *G. caledonium* (GC) had positive effects on the AMF colonization of *Bt* maize or non-*Bt* maize. This, in turn, resulted in higher yields of *Bt* maize and non-*Bt* maize, and the cultivation of *Bt* maize did not significantly affected AMF colonization. Moreover, *Bt* maize had marked adverse effects on the food utilization, the reproduction, growth, and development of *M. separata*, particularly in combination with GC inoculation. Furthermore, GC inoculation was viable for *Bt* maize in their defense against larvae due to its ability to reduce their food utilization ability, which negatively affects the reproduction, growth, and development of *M. separata*. Simultaneously, the GC inoculation had adverse effects on the production of non-*Bt* maize due to the high potential risk of population occurrence through the enhancing of their food utilization ability, and positively affecting the reproduction, growth, and development of *M. separata*. The results indicated that the AMF inoculation of GC was conducive to improving the performance of *Bt* maize for the *M. separata* control, and it was also a very friendly and effective way for increasing the yield and reducing fertilizer use of crop plants. Therefore, we believe that AMF will play important ecological functions in the future *Bt* maize ecosystem.

##  Supplemental Information

10.7717/peerj.7679/supp-1 Data S1 Raw data

## Additional Information and Declarations

### Data Availability

The following information was supplied regarding data availability: The raw measurements are available in File S1 and includes associated raw values and four figures (Figs. 1–4).
